# Drought stress induced by polyethylene glycol (PEG) in local maize at the early seedling stage

**DOI:** 10.1016/j.heliyon.2023.e20209

**Published:** 2023-09-16

**Authors:** Novilda Elizabeth Mustamu, Koko Tampubolon, Alridiwirsah, Mohammad Basyuni, Duraid K.A. AL-Taey, Haider Jawad Kadhim AL Janabi, Mohammad Mehdizadeh

**Affiliations:** a Universitas Labuhanbatu, Faculty of Science and Technology, Program Study of Agrotechnology, Rantauprapat 21415, Sumatera Utara, Indonesia; b Universitas Sumatera Utara, Faculty of Agriculture, Program Study of Agrotechnology, Medan 20155, Indonesia; c Universitas Muhammadiyah Sumatera Utara, Faculty of Agriculture, Program Study of Agrotechnology, Medan 20238, Indonesia; d Universitas Sumatera Utara, Faculty of Forestry, Program Study of Forestry, Medan 20155, Indonesia; e Department of Horticulture, AL-Qasim Green University, Babylon, Iraq; f Medical Laboratories Techniques Department / AL-Mustaqbal University College, 51001 Hillah, Babil, Iraq; g Department of Agronomy and Plant Breeding, Faculty of Agriculture and Natural Resources, University of Mohaghegh Ardabili, Ardabil, Iran

**Keywords:** Maize, Polyethylene glycol, Germination, Drought stress, Root histology

## Abstract

Drought stress adversely impacts growth, crop production, reproductive organ development, and yield characteristics in maize. As a drought-sensitive crop, maize (*Zea mays* L.) shows considerable varietal differences. A study was conducted at the Tissue Culture Laboratory, Department of Biology, Faculty of Mathematics and Natural Sciences, University of North Sumatra, Medan, Indonesia in order to identify drought-tolerant maize varieties. During germination and early seedling growth, 16 local accessions were evaluated for drought tolerance. Based on local climate and soil conditions, these specific accessions were chosen. The varieties were tested against five levels of drought stress imposed by Polyethylene glycol 6000 (PEG-6000) at 10, 20, 30, 40, and 50%. An experiment with three replications was conducted using a completely randomized design. In the study, local maize accessions (BI3, SB5, DS2, and MN3) and the hybrid variety (H) showed the capability of tolerating drought stress. Generally, germination time, germination percent and vigor index, root and shoot length, shoot ratio, and fresh and dry weight were decreased by increasing PEG concentrations (up to 50%). According to ANOVA results, shoot water content was not significantly affected by the PEG, nor was the interaction between the PEG and the accessions. The root water content, however, was significantly affected by PEG, and the interaction between PEG and accessions. Although interactions between accessions with low PEG concentrations improved germination characteristics, the root histology of the accessions varied. According to drought tolerance indexes, five maize accessions are drought-tolerant, including H (0.683), SB5 (0.617), DS2 (0.565), MN3 (0.512), and BI3 (0.504). The drought-tolerant varieties are recommended in regions with low rainfall or low water sources since they are less water-intensive and produce higher yields.

## 1 Introduction

Indonesia is one of the leading maize suppliers in the Southeast Asian region. The majority of maize production in Indonesia is sourced from small-scale farmers. The country has a vast potential for increasing maize yields and improving crop productivity. Increased crop yield will benefit both farmers and the local economy. Indonesian maize fields are established in arid areas, which is inappropriate for rice or vegetable farming [1]. Due to this land condition, maize yield is extremely limited. Therefore, increasing maize yield and quality is essential for Indonesia to become a significant player in the corn market [2]. Modern technology and infrastructure investment are needed to ensure a sustainable future for the country's maize industry and food security. The government is implementing a series of initiatives to increase maize production to address this issue. These include the use of improved varieties, better irrigation techniques, and fertilizer application. Additionally, research initiatives are being conducted to find new ways to increase maize yields and reduce the risks associated with farming in arid areas.

North Sumatra is the sixth largest maize producing center in Indonesia after West Java, West Sumatra, West Nusa Tenggara, South Sumatra, and Jambi in 2020 [3]. The establishment of these centers is essential to support food self-sufficiency, but it is inappropriate to optimize maize productivity without considering genetic and environmental factors. Statistics of Sumatera Utara [4] reported a decrease in the harvested area of maize plants by 47,481 ha (14.78%) in 2021. It was also discovered that uncertain climate change and shifting seasons will negatively impact cultivated plants' ecological suitability [5]. Dai [6] added that climate change is associated with drought frequency increases. According to Mi et al. [7], drought stress can cause maize seed production losses ranging from 40 to 65% depending on genotype, plant growth stage, intensity, and duration.

Drought-tolerant plants are needed in arid regions to prevent maize yield losses. Farmers in Indonesia continue to use local maize varieties for replanting to cut production costs. Currently, 80–90% of Indonesian varieties are hybrid maizes, and only 10–20% are local varieties [2]. Although these varieties have a low yield, they have several advantages such as earlier flowering (anthesis, silking) and faster harvest time compared to hybrid and composite varieties [8]. The high cost of hybrid maize seeds also forces farmers to select local varieties, especially in North Sumatra, according to Amzeri [9]. The local varieties are also believed to be more resilient to climate change than hybrid varieties. Hence, it is important to conserve these varieties to ensure food security in the future.

Polyethylene glycol (PEG-6000) treatment can be applied to identify local maize varieties as sensitive or tolerant to drought stress before germination [10,11]. This method can be used to identify the best varieties for drought-prone areas and help in conservation efforts. Additionally, it can also be used to inform breeding programs to develop drought-resistant varieties for the future. According to Verslues et al. [12], PEG-6000 is the most effective water potential control agent and is non-uptakeable by plants. Furthermore, drought stress can produce reactive oxygen species (ROS) and break down unsaturated fatty acids, which cause structural degradation of seeds and inhibit germination [13]. Fenta et al. [14] reported that drought stress will cause changes in root anatomy, such as an increase in cortex diameter due to plant adaptation forms. A review of previous studies revealed differences in maize plant responses to PEG dosages. The most notable differences were observed in the shoot and root biomass of maize plants grown in mediums with varying PEG concentrations. PEG concentrations could act as a limiting factor by affecting maize plant growth during the germination and seedling stages, according to previous studies [[15], [16], [17], [18]].

Drought-tolerant maize varieties from North Sumatra have not been discovered and drought stress's effect on root anatomy has not been studied. In this regard, this study examined several drought-tolerant local maize accessions during the germination phase in North Sumatra and determined their effect on root histology after treatment with PEG-6000. Our research questions were to investigate the response of selected maize accessions to PEG-induced drought stress and to identify accessions that are tolerant to drought stress. We also hypothesized that some accessions may be more tolerant to drought stress than others.

## 2 Materials and methods

### 2.1 Seed selection and source of local maize accessions

**Fig. 1:**
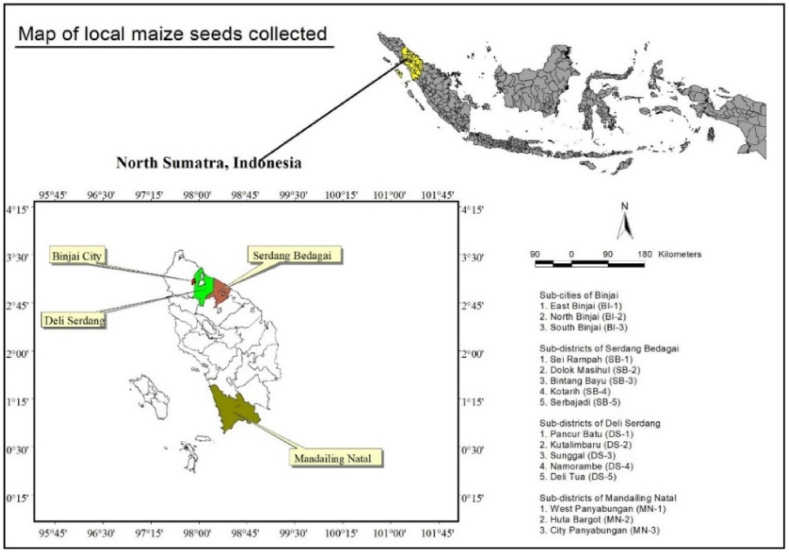
A map of the area in North Sumatra where local maize seed was collected. In North Sumatra Province, Indonesia, local maize accessions were collected in Binjai City, Serdang Bedagai, Deli Serdang District, and Mandailing Natal District.

Seeds were generally selected based on their size, shape, color, and texture. A quality seed selection process was used to select seeds that would be appropriate for drought stress experiments. The process involved visual assessments of the seed's shape and size, as well as microscopic evaluations of the seed's surface texture. Using the history of cultivation in different regions as a guide, the chosen seeds were confirmed to have an acceptable germination rate and the ability to survive drought conditions. Moreover, local accessions from different regions were selected based on farmer preferences and their adaptability to environmental conditions and their productivity under biotic and abiotic stresses. There were three different local maize accessions collected from Binjai City, five accessions from Serdang Bedagai, five accessions from Deli Serdang District, and three accessions from Mandailing Natal District, North Sumatra Province, Indonesia (Fig. 1). To provide a comparison basis, a hybrid maize variety (BISI-79) was provided by BISI International Ltd. There were 10 cobs taken from each accession that met physiological criteria, with brown husks and yellow seeds. Seeds were dried until their moisture content reached 14%.

### 2.2 Experimental design

Germination and histological analysis of maize were carried out at the Tissue Culture Laboratory, Department of Biology, Faculty of Mathematics and Natural Sciences, University of North Sumatra, Medan. During July to November 2022, local maize seeds were collected for germination. A completely randomized design was selected with two factors and three replications. The first factor was local maize accessions (BI1; BI2; BI3; SB1; SB2; SB3; SB4; SB5; DS1; DS2; DS3; DS4; DS5; MN1; MN2; and MN3) and a hybrid variety (BISI-79) is regarded as a control treatment for accessing drought stress impacts on these accessions. In the second factor, different concentrations of PEG-6000 (0; 10; 20; 30; 40; and 50%) were used to simulate drought stress.

### 2.3 PEG-6000 treatment

A total of 100, 200, 300, 400, and 500 g of PEG-6000 were weighed and dissolved in 1 L of distilled water until homogeneous. After placing the filter paper on top of the Petri dish, dripping PEG-6000 solution was applied until the condition was moist. The seeds were placed in Petri dishes with corresponding PEG concentrations (0, 10 20, 30, 40, and 50%). A total of 50 seeds were counted from each accession, soaked in the PEG solutions for 2 h, and the seeds were transferred to the filter paper. The solutions were applied on a daily basis after draining the previous day's solution for each treatment for 14 days. Following this, a temperature of 28.7 °C and 65% humidity were recorded in the laboratory.

### 2.4 Data collection and processing

There were several germination characteristics observed in this study, including the number and percent of germination, the vigor index, the length of roots and shoots, the root: shoot ratio, fresh and dry weights, and water content. We also examined root histological characteristics, such as epidermis, cortex, and stele, and calculated heritability and drought tolerance indexes. The number of seeds germinating up to 14 DAS with an interval of 2 d was counted. Based on Scott et al. [19], equations (1), (2)) were used to estimate germination percent and vigor index. Equation (3) was used to calculate water content based on fresh and dry weight.

At the end of the study, the root and shoot were separated, and each parameter was measured accordingly. Dry weight was determined by drying the root and shoot for 48 h at 80 °C [20]. In addition, a transverse incision was made for measuring root histology using the paraffin method [21] and images were taken with the Axiovision 4.8 application at a magnification of 10 × 10.

(1) $$\text{Germination}\;\text{percent}=\frac{\text{Number}\;\text{of}\;\text{germinated}\;\text{seeds}}{\text{Number}\;\text{of}\;\text{seeds}}\times 100\%$$

(2) Vigorindex=germinationpercent×seedlinglength(root+shoot)

(3) $$Water\;content=\frac{\text{Fresh}\;\text{weight}-\text{dry}\;\text{weight}}{\text{Fresh}\;\text{weight}}\times 100\%$$

(4) $$\sigma^{2}g=\frac{\text{Mean}\;\text{of}\;\text{Square}\;\left(\text{MS}\right)\;\text{at}\;\text{accession}-\text{MS}\;\text{error}}{\text{replicates}}$$

(5) $$\sigma^{2}p=\sigma^{2}g+\sigma^{2}e\;\left(\text{MS}\;\text{error}\right)$$

(6) $$\text{GCV}=\frac{\sqrt{\sigma^{2}g}}{\bar{X}}\times 100\%$$

(7) $$\text{PCV}=\frac{\sqrt{\sigma^{2}p}}{\bar{X}}\times 100\%$$

(8) $$h^{2}=\frac{\sigma^{2}g}{\sigma^{2}p}$$

(9) $$\text{DTI}=1-\frac{\text{Root}\;\text{dry}\;\text{weight}\;\text{in}\;\text{PEG}-\text{treated}}{\text{Root}\;\text{dry}\;\text{weight}\;\text{in}\;\text{untreated}}$$

Calculations of genotypic (σ^2^g) and phenotypic (σ^2^p) variance, genotypic and phenotypic coefficients of variation (GCV and PCV), as well as heritability (h^2^), are presented in equations (4), (6), (7), (8)). The heritability value (h^2^) were classified as high, moderate, and low when h^2^> 50%; ≥20–50%, and <20%, respectively [22]. Based on the root dry weight, the drought tolerance index (DTI) was calculated (equation (9)), where the highest DTI value indicates drought tolerance [23].

### 2.5 Statistical analysis

IBM SPSS software was used to analyze the collected data using Completely Randomized Design. In this study, data analysis was performed using analysis of variance (ANOVA) and Duncan's Multiple Range Test (DMRT), and P < 0.05 was considered significant.

## 3 Results

### 3.1 Germination speed (seeds/days)

**Fig. 2:**
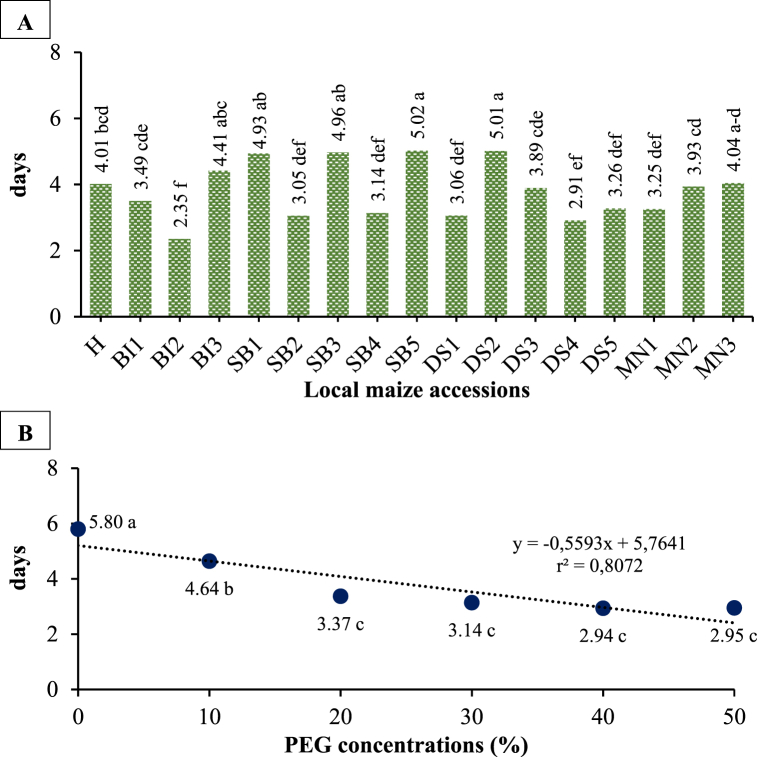
The germination speed in local maize from studied accessions (A) and PEG concentrations (B). Differences between means for variables with the same letter are not statistically significant according to DMRT test.

Local maize accessions and PEG concentrations significantly affected germination speed, but their interaction had no significant effect (Fig. 2(A and B) and Table 1, Table 2). In comparison to other accessions, SB5 and DS2 germinated faster at 5.02 and 5.01 days, respectively. Germination speed decreased by 49.14% along with an increase in PEG concentration of 50%.

**Table 1:** Analysis of variance (ANOVA) of observed factors in maize accessions under drought stress. The ANOVA was used to determine the difference in the mean values of observed factors for maize accessions under drought stress.

Factors	Source	Sum of Squares	df	Mean Square	F	Significant
**Speed of Germination**	Block	1.762	2	0.881	0.510 ns	0.601
	Accessions (A)	201.695	16	12.606	7.296 *	0.000
	PEG (P)	346.040	5	69.208	40.056 *	0.000
	A x P	137.504	80	1.719	0.995 ns	0.500
	Error	349.015	202	1.728		
	Total	1036.016	305			
**Germination Percent**	Block	57.595	2	28.797	0.110 ns	0.896
	Accessions (A)	47244.549	16	2952.784	11.252 *	0.000
	PEG (P)	22899.242	5	4579.848	17.452 *	0.000
	A x P	16594.980	80	207.437	0.790 ns	0.886
	Error	53009.072	202	262.421		
	Total	139805.438	305			
**Vigor Index**	Block	59.171	2	29.585	0.580 ns	0.561
	Accessions (A)	16343.911	16	1021.494	20.032 *	0.000
	PEG (P)	11890.299	5	2378.060	46.635 *	0.000
	A x P	2732.262	80	34.153	0.670 ns	0.980
	Error	10300.591	202	50.993		
	Total	41326.235	305			
**Root Length**	Block	52.206	2	26.103	1.907 ns	0.151
	Accessions (A)	1442.291	16	90.143	6.585 *	0.000
	PEG (P)	1563.548	5	312.710	22.842 *	0.000
	A x P	656.244	80	8.203	0.599 ns	0.995
	Error	2765.381	202	13.690		
	Total	6479.669	305			
**Shoot Length**	Block	5.926	2	2.963	0.248 ns	0.781
	Accessions (A)	3702.700	16	231.419	19.330 *	0.000
	PEG (P)	1606.846	5	321.369	26.843 *	0.000
	A x P	460.764	80	5.760	0.481 ns	1.000
	Error	2418.367	202	11.972		
	Total	8194.603	305			
**Root: Shoot Ratio**	Block	0.222	2	0.111	1.794 ns	0.169
	Accessions (A)	7.555	16	0.472	7.620 *	0.000
	PEG (P)	0.191	5	0.038	0.617 ns	0.687
	A x P	1.434	80	0.018	0.289 ns	1.000
	Error	12.516	202	0.062		
	Total	21.918	305			
**Roots Fresh Weight**	Block	24.474	2	12.237	0.131 ns	0.877
	Accessions (A)	74776.867	16	4673.554	50.155 *	0.000
	PEG (P)	25716.385	5	5143.277	55.196 *	0.000
	A x P	33335.849	80	416.698	4.472 *	0.000
	Error	18822.827	202	93.182		
	Total	152676.402	305			
**Shoots Fresh Weight**	Block	1.060	2	0.530	0.008 ns	0.992
	Accessions (A)	9704.081	16	606.505	9.009 *	0.000
	PEG (P)	6349.831	5	1269.966	18.864 *	0.000
	A x P	5852.898	80	73.161	1.087 ns	0.318
	Error	13599.248	202	67.323		
	Total	35507.118	305			
**Roots Dry Weight**	Block	90.845	2	45.423	2.032 ns	0.134
	Accessions (A)	2868.160	16	179.260	8.020 *	0.000
	PEG (P)	12973.390	5	2594.678	116.079 *	0.000
	A x P	2577.762	80	32.222	1.442 *	0.021
	Error	4515.261	202	22.353		
	Total	23025.418	305			
**Shoots Dry Weight**	Block	79.065	2	39.533	3.726 *	0.026
	Accessions (A)	1556.778	16	97.299	9.171 *	0.000
	PEG (P)	1004.480	5	200.896	18.936 *	0.000
	A x P	714.168	80	8.927	0.841 ns	0.811
	Error	2143.013	202	10.609		
	Total	5497.504	305			
**Roots Water Content**	Block	120.532	2	60.266	0.511 ns	0.601
	Accessions (A)	12881.214	16	805.076	6.830 *	0.000
	PEG (P)	9410.023	5	1882.005	15.966 *	0.000
	A x P	21604.388	80	270.055	2.291 *	0.000
	Error	23810.652	202	117.875		
	Total	67826.809	305			
**Shoots Water Content**	Block	1784.980	2	892.490	4.173 *	0.017
	Accessions (A)	20033.997	16	1252.125	5.854 *	0.000
	PEG (P)	800.422	5	160.084	0.748 ns	0.588
	A x P	17417.560	80	217.720	1.018 ns	0.451
	Error	43204.249	202	213.882		
	Total	83241.208	305			
**Root Epidermis**	Block	13665.543	2	6832.771	202.484 *	0.000
	Accessions (A)	35307.544	16	2206.721	65.394 *	0.000
	PEG (P)	3918.524	5	783.705	23.224 *	0.000
	A x P	66051.012	80	825.638	24.467 *	0.000
	Error	6816.452	202	33.745		
	Total	125759.075	305			
**Cortex**	Block	1000192.707	2	500096.354	574.659 *	0.000
	Accessions (A)	1137326.770	16	71082.923	81.681 *	0.000
	PEG (P)	116697.566	5	23339.513	26.819 *	0.000
	A x P	2096495.377	80	26206.192	30.113 *	0.000
	Error	175790.135	202	870.248		
	Total	4526502.554	305			
**Stele**	Block	10869487.858	2	5434743.929	792.525 *	0.000
	Accessions (A)	2520689.834	16	157543.115	22.974 *	0.000
	PEG (P)	269657.098	5	53931.420	7.865 *	0.000
	A x P	7015251.343	80	87690.642	12.788 *	0.000
	Error	1385215.333	202	6857.502		
	Total	22060301.466	305			

ns, and *: Not significant and significant at 5% of probability levels, respectively.

**Table 2:** Interaction of accessions with PEG concentrations on germination and histology of hybrid (H) and local maize from Binjai City.

Maize accessions	PEG conc. (%)	SG (seed/days)	GP (%)	VI	RL (cm)	SL (cm)	FW (g)		DW (g)		WC (%)		HT (μm)		
							Root	Shoot	Root	Shoot	Root	Shoot	Root epidermis	Cortex	Stele
H	0	7.48	79.33	34.50	23.87	19.80	56 g-x	30.42	30a-i	11	46rst	63	15x-ae	152ae-ap	287ad-al
	10	4.16	60.67	25.01	22.13	19.23	64b-u	29.66	15f-s	11	76a-n	62	22n-ae	198u-ao	275ae-al
	20	4.48	60.00	23.45	22.03	18.43	45n-z	33.08	10m-s	10	77a-n	69	37h-w	204t-ao	522j-w
	30	2.58	38.67	14.01	17.80	18.33	57f-x	17.47	9o-s	10	83a-f	41	11aa-ae	142ah-aq	251ah-al
	40	2.54	36.00	11.91	15.63	18.00	26w-z	14.87	8o-s	9	57k-t	32	12z-ae	134ai-aq	224aj-am
	50	2.84	42.67	13.68	14.00	17.60	41o-z	23.00	4s	8	90a	61	55c-h	284i-z	755c-g
BI1	0	5.71	52.67	23.76	18.10	27.67	61c-v	28.92	27b-l	11	55m-t	59	6adae	99ao-ar	259 ag-al
	10	4.52	50.67	20.31	15.20	24.20	26w-z	25.08	18c-s	10	11u	56	35h-x	311f-s	424s-ag
	20	1.85	34.00	12.62	15.07	23.17	38q-z	26.94	18c-s	8	47q-t	61	20r-ae	171 ab-ap	176akalam
	30	2.45	32.00	11.81	13.97	22.60	32t-z	23.03	15f-s	6	41t	54	48 d-k	306f-t	723c-h
	40	3.15	43.33	14.54	13.10	20.37	48m-z	19.00	10m-s	6	73a-n	55	24 l-ae	256 l-af	771cde
	50	3.25	45.33	13.80	11.67	19.23	56h-x	13.91	7p-s	4	83a-f	61	93a	460b	958a
BI2	0	4.41	52.00	21.18	15.80	25.03	80a-n	33.17	16f-s	10	77a-m	69	16u-ae	314e-s	475n-aa
	10	3.14	45.33	17.61	14.77	23.30	33s-z	25.79	14 g-s	8	57 l-t	63	18s-ae	404b-g	474n-aa
	20	1.36	21.33	7.74	13.53	22.20	41o-z	12.08	11j-s	7	72a-n	35	20r-ae	323e-q	446o-ad
	30	1.60	24.67	8.81	13.87	21.73	41o-z	23.35	9o-s	7	77a-m	65	37 g-u	389b-i	490m-y
	40	1.46	29.33	9.88	12.77	21.17	24 xyz	17.58	7p-s	6	71a-n	60	24 l-ae	288h-x	482m-z
	50	2.11	34.00	10.75	10.67	20.93	27v-z	16.57	4rs	6	82a-f	59	26 l-ae	332e-p	519j-w
BI3	0	7.55	68.00	35.32	21.57	28.17	69b-r	29.73	31a-f	11	54n-t	58	80 ab	328e-p	575h-t
	10	6.22	73.33	32.31	20.33	24.73	81a-m	36.80	19c-s	10	76a-n	67	26 l-ae	191v-ao	297ac-al
	20	3.80	54.00	24.94	20.97	24.77	51 l-z	26.62	18c-s	9	62f-t	65	65b-e	230p-ak	476n-aa
	30	3.68	49.33	20.14	18.30	23.20	31t-z	24.39	16f-s	8	48p-t	64	13 y-ae	155ad-ap	298 ab-al
	40	2.42	38.00	15.72	18.50	22.90	36r-z	20.22	13j-s	8	63e-s	56	35h-x	251n-ag	481m-z
	50	2.77	58.67	21.78	16.27	21.10	20z	31.01	10 l-s	7	47q-t	74	33i-y	282j-z	513k-w

(a) Table 2. Interaction of accessions with PEG concentrations on germination and histology of local maize from Serdang Bedagai.															
Maize accessions	PEG conc. (%)	SG (seed/days)	GP (%)	VI	RL (cm)	SL (cm)	FW (g)		DW (g)		WC (%)		HT (μm)		
							Root	Shoot	Root	Shoot	Root	Shoot	Root epidermis	Cortex	Stele
SB1	0	7.45	86.67	41.50	23.27	24.50	90a-g	48	31a-g	11	64 d-r	76	22n-ae	169 ab-ap	411t-ai
	10	5.57	77.33	35.23	21.53	24.13	89a-h	38	23b-p	11	74a-n	70	21q-ae	199u-ao	431s-af
	20	4.67	80.00	34.79	21.60	22.20	80a-m	38	22c-p	11	72a-n	70	20r-ae	154ad-ap	490m-y
	30	3.70	70.67	28.33	19.33	20.50	70b-r	30	18c-s	11	73a-n	51	5ae	198u-ao	386v-aj
	40	4.04	68.67	25.12	17.97	18.73	52j-z	36	11k-s	5	78a-l	85	18t-ae	154ad-ap	192akalam
	50	4.17	68.67	22.72	15.70	17.27	77a-n	32	8o-s	4	89 ab	84	15x-ae	162ac-ap	403u-ai
SB2	0	3.32	54.67	23.28	21.80	18.53	91a-f	32	33a-e	11	63f-t	63	20r-ae	184x-ao	337x-ak
	10	4.37	81.33	27.31	18.77	15.30	85a-k	42	21c-r	10	75a-n	74	17t-ae	182z-ap	314z-al
	20	2.87	49.33	16.42	16.37	16.00	65b-t	41	17e-s	9	70a-o	77	43f-o	380b-j	667 d-l
	30	3.25	60.67	18.60	15.57	14.27	64b-u	28	16e-s	8	73a-n	70	24m-ae	412b-f	705c-i
	40	2.92	52.00	13.94	14.03	12.83	61c-v	37	16f-s	6	73a-n	79	30j-ab	451bcd	756c-g
	50	1.56	29.33	8.12	13.43	13.77	59 d-x	14	13i-s	6	77a-n	58	50 d-j	108an-ar	513k-w
SB3	0	7.56	89.33	43.97	21.93	27.30	68b-s	45	24b-o	11	63e-s	74	42f-p	379b-k	932 ab
	10	6.72	88.67	41.91	21.43	26.00	50 l-z	43	17 d-s	10	65c-r	76	37 g-v	308f-t	984a
	20	4.21	70.67	32.84	20.70	25.43	58e-x	30	13j-s	9	77a-m	67	25 l-ae	148 ag-ap	434r-af
	30	4.05	77.33	34.76	19.50	24.83	55h-y	32	13i-s	8	75a-n	74	24m-ae	168 ab-ap	403u-ai
	40	4.14	72.00	31.55	17.77	25.97	65b-u	34	16f-s	7	74a-n	77	23n-ae	37aqar	402u-ai
	50	3.09	46.00	18.16	16.00	24.43	53i-z	21	14i-s	5	73a-n	70	52c-i	361b-l	852 abc
SB4	0	5.86	71.33	28.13	19.83	19.67	75a-o	38	30a-h	12	59 g-t	65	18s-ae	148 ag-ap	305 ab-al
	10	3.76	58.67	19.25	16.30	15.43	80a-m	26	20c-s	11	74a-n	55	17t-ae	257 l-ae	454o-ad
	20	2.86	44.00	13.05	16.27	14.97	86a-j	25	18c-s	9	78a-l	60	10aa-ae	261 l-ac	467o-ab
	30	2.38	42.00	12.82	15.97	14.50	71a-q	18	16f-s	4	77a-m	69	11aa-ae	135ai-aq	443p-ae
	40	2.10	36.67	10.33	14.73	13.27	68b-s	19	12j-s	3	81a-h	79	10 ab-ae	112am-ar	436q-ae
	50	1.87	28.67	7.37	13.40	12.30	61c-v	18	11j-s	3	80a-i	78	7acadae	151af-ap	267af-al
SB5	0	6.93	98.00	55.89	26.00	31.00	91a-f	45	46a	12	49o-t	72	24 l-ae	252n-ag	501 l-y
	10	6.60	88.67	46.37	23.30	29.07	89a-h	40	27b-k	11	68a-q	71	43f-n	295h-v	523j-w
	20	4.42	83.33	39.86	20.10	27.87	78a-n	39	22c-p	11	70a-o	71	58c-f	332e-p	460o-ac
	30	4.53	81.33	35.66	19.87	24.27	80a-m	37	16e-s	10	78a-l	72	41f-q	236p-ai	443p-ae
	40	4.09	66.67	25.93	17.87	23.73	65b-t	31	14h-s	9	78a-l	61	21p-ae	288h-y	481m-z
	50	3.54	64.00	23.75	16.40	21.20	60 d-w	34	9o-s	8	84a-f	71	52c-i	320e-r	478m-z

(b) Table 2. Interaction of accessions with PEG concentrations on germination and histology of local maize from Deli Serdang.															
Maize accessions	PEG conc. (%)	SG (seed/days)	GP (%)	VI	RL (cm)	SL (cm)	FW (g)		DW (g)		WC (%)		HT (μm)		
							Root	Shoot	Root	Shoot	Root	Shoot	Root epidermis	Cortex	Stele
DS1	0	5.42	66.67	30.56	20.83	24.33	87a-i	39	21c-r	15	74a-n	59	42f-o	349c-n	475n-aa
	10	2.45	54.00	23.81	18.90	23.30	83a-l	26	19c-s	5	76a-n	82	37 g-f	273k-ab	477n-aa
	20	2.35	46.67	19.28	18.83	22.80	77a-n	25	15f-s	2	77a-m	88	55c-h	383b-j	803bcd
	30	2.29	41.33	16.47	18.47	22.10	72a-q	25	19c-s	3	73a-n	85	26 l-ad	294h-w	564h-u
	40	2.33	42.00	15.42	16.63	20.57	49 l-z	16	7p-s	1	84a-f	88	25 l-ae	346 d-o	521j-w
	50	3.50	61.33	20.18	13.77	18.47	40p-z	30	16f-s	5	58h-t	84	33i-z	454BCE	582h-t
DS2	0	5.50	84.00	51.50	28.13	32.60	95 abc	45	39 ab	11	58i-t	72	21p-ae	176aa-ap	297ac-al
	10	6.26	76.00	40.75	25.33	27.70	92a-d	47	21c-r	15	76a-n	66	43f-o	734a	590 g-s
	20	3.16	65.33	31.16	22.13	25.53	91a-e	36	20c-s	13	76a-n	61	40f-r	253n-ag	507k-x
	30	5.25	70.00	31.29	19.90	24.47	88a-h	30	17 d-s	10	79a-k	64	68bcd	249n-ag	694c-i
	40	4.48	71.33	28.51	18.70	21.60	86a-j	33	15f-s	13	81a-g	57	43f-n	273k-ab	710c-i
	50	5.39	78.00	27.09	14.90	19.40	82a-m	48	10o-s	11	87 abc	77	71BCE	322e-q	921 ab
DS3	0	6.29	66.67	31.11	23.53	23.10	69b-r	35	22c-p	4	68a-q	88	22p-ae	237p-ai	319z-ak
	10	4.42	58.00	21.23	17.23	19.53	80a-m	25	21c-q	7	72a-n	69	58c-g	331e-p	606e-q
	20	3.19	55.33	19.38	17.53	16.77	51k-z	30	12j-s	2	76a-n	89	43f-n	417b-e	647 d-m
	30	3.59	50.67	17.61	17.27	17.40	30u-z	21	9o-s	2	68a-q	87	38f-s	390b-h	613e-p
	40	3.44	47.33	16.23	16.80	16.23	38q-z	21	7o-s	2	79a-l	86	31j-aa	243o-ah	639 d-n
	50	2.43	37.33	12.32	16.23	16.83	20yz	18	5qrs	2	75a-n	83	22n-ae	182 y-ap	592f-s
DS4	0	3.30	43.33	20.05	19.17	27.97	56h-x	24	32a-f	9	42nd	67	19s-ae	258 l-ad	505k-x
	10	3.27	50.67	21.23	18.70	23.60	92a-e	26	28b-j	8	69a-p	69	20r-ae	259 l-ad	417 l-ah
	20	4.34	60.00	23.98	18.63	20.87	70b-r	32	18c-s	7	73a-n	76	28k-ac	255m-af	604f-r
	30	2.57	56.00	20.04	16.60	20.07	59 d-w	25	13h-s	2	76a-n	87	44f-m	347 d-o	617e-o
	40	2.33	40.67	12.87	14.30	17.60	98 ab	20	12j-s	2	86a-d	88	23n-ae	77apaqar	97am
	50	1.65	32.00	9.00	12.80	15.90	88a-h	14	12j-s	2	86a-e	82	14 y-ae	279j-aa	497m-y
DS5	0	4.27	71.33	37.18	24.47	27.17	81a-m	32	34a-d	7	58j-t	77	35h-x	306f-t	672 d-k
	10	3.84	62.67	31.27	23.33	27.23	71a-q	26	22b-p	3	67b-r	87	22o-ae	22ar	690 d-i
	20	3.59	50.67	22.16	19.97	23.50	74a-p	26	21c-r	8	70a-o	65	16v-ae	128aj-aq	417t-ah
	30	2.37	46.67	19.23	19.13	21.70	77a-n	27	16f-s	4	78a-l	85	35h-x	180z-ap	391v-ai
	40	2.85	56.00	21.55	17.13	20.20	53i-z	26	13h-s	6	74a-n	72	27k-ac	121 al-ar	556i-v
	50	2.67	55.33	18.66	15.17	18.43	57e-x	22	13h-s	3	73a-n	82	14x-ae	172 ab-ap	474n-aa

(c) Table 2. Interaction of accessions with PEG concentrations on germination and histology of local maize from Mandailing Natal.															
Maize accessions	PEG conc. (%)	SG (seed/days)	GP (%)	VI	RL (cm)	SL (cm)	FW (g)		DW (g)		WC (%)		HT (μm)		
							Root	Shoot	Root	Shoot	Root	Shoot	Root epidermis	Cortex	Stele
MN1	0	6.17	72.67	25.46	10.90	24.13	83a-l	36	27b-m	8	67b-r	75	28k-ac	288h-x	682 d-j
	10	4.23	56.00	21.11	13.03	24.67	74a-p	25	19c-s	7	74a-n	72	16w-ae	253n-ag	437q-ae
	20	3.10	44.00	18.13	17.27	24.00	73a-p	27	16e-s	4	77a-n	82	8acadae	205t-an	377w-aj
	30	2.45	33.33	13.75	17.23	24.53	49 l-z	25	13h-s	3	72a-n	84	12aa-ae	215r-am	298 ab-al
	40	1.75	26.00	9.93	15.87	22.53	55h-x	12	13j-s	3	76a-n	69	24m-ae	209s-an	248ai-am
	50	1.83	30.00	11.25	16.17	21.27	38q-z	14	11j-s	2	70a-o	84	19s-ae	194u-ao	362w-aj
MN2	0	5.70	68.00	34.23	21.17	29.17	98 ab	33	27b-k	10	71a-o	69	23n-ae	233p-aj	684 d-j
	10	4.43	57.33	25.76	19.10	25.93	96 ab	30	22c-p	8	77a-n	74	45e-l	360b-m	730c-h
	20	3.17	43.33	17.57	18.23	22.60	96 ab	27	15f-s	6	83a-f	75	96a	299 g-u	594f-s
	30	3.48	48.67	18.17	17.30	20.57	89a-h	28	14 g-s	6	83a-f	67	28k-ac	282j-z	702c-i
	40	2.62	42.00	15.53	16.67	19.63	80a-m	24	20c-s	6	75a-n	69	84 ab	222q-al	758c-f
	50	4.18	52.00	17.18	14.70	18.93	69b-r	22	13i-s	6	80a-i	68	13 y-ae	181z-ap	331 y-ak
MN3	0	5.67	70.00	38.63	24.73	30.80	105a	36	35 abc	13	66b-r	65	16v-ae	127ak-ar	153 alam
	10	4.93	70.00	34.30	21.60	28.20	98 ab	34	24b-n	12	74a-n	60	27k-ac	112am-a	308aa-al
	20	3.88	57.33	26.21	18.40	27.63	89a-h	27	19c-s	11	78a-l	59	26 l-ad	188x-ao	485m-z
	30	3.16	48.67	21.39	16.97	27.43	88a-h	29	17e-s	10	80a-j	64	14x-ae	189w-ao	507k-x
	40	3.32	48.67	20.98	16.47	26.73	78a-n	22	15f-s	6	79a-k	67	6adae	187x-ao	434r-af
	50	3.28	46.00	18.40	15.03	25.03	74a-p	23	8o-s	6	88 ab	73	12aa-ae	208s-an	494m-y

Note: Means with the same letters are not significantly different based on Duncan's test at the 5% of probability level. SG = germination speed; GP = germination percent; VI = vigor index; RL = root length; SL = shoot length; FW = fresh weight; DW = dry weight; WC = water content; HT = histological tissue.

### 3.2 Germination percent and vigor index

**Fig. 3:**
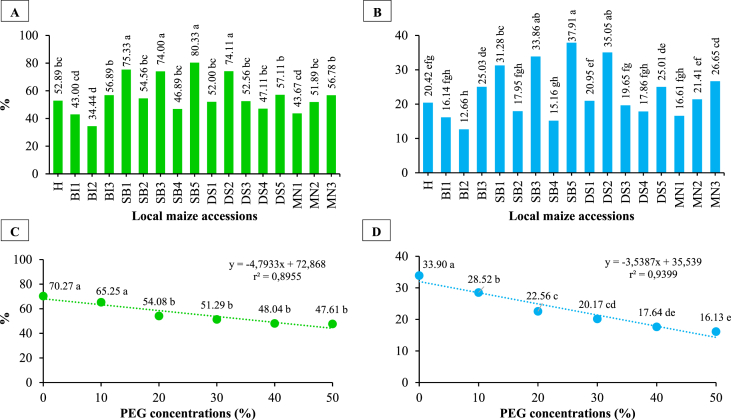
Germination percent and vigor index of local maize from several accessions (A, B) and PEG concentrations (C, D). Differences between means for variables with the same letter are not statistically significant according to DMRT test.

There was a significant effect of local maize accessions and PEG concentrations on germination percent and vigor index. However, their interaction had no significant effect (Fig. 3(A-D) and Table 1, Table 2). With an increase in PEG concentration, germination percent and vigor index decreased. This trend was observed in all four regions studied. Due to 10% PEG, the germinated seeds of local maize had increased to 3.65 and were inhibited by a 50% PEG concentration. When compared with other accessions, SB5 had the highest germination percentage and vigor index of 80.33% and 37.91, respectively. As shown in Table 2, SB3 was the most affected accessions by the highest concentration of PEG (50%). There was the greatest reduction in germination percent and vigor index due to higher PEG concentrations (50%) in SB3, with 44.33 and 25.81% respectively.

### 3.3 Root and shoot length (cm)

**Fig. 4:**
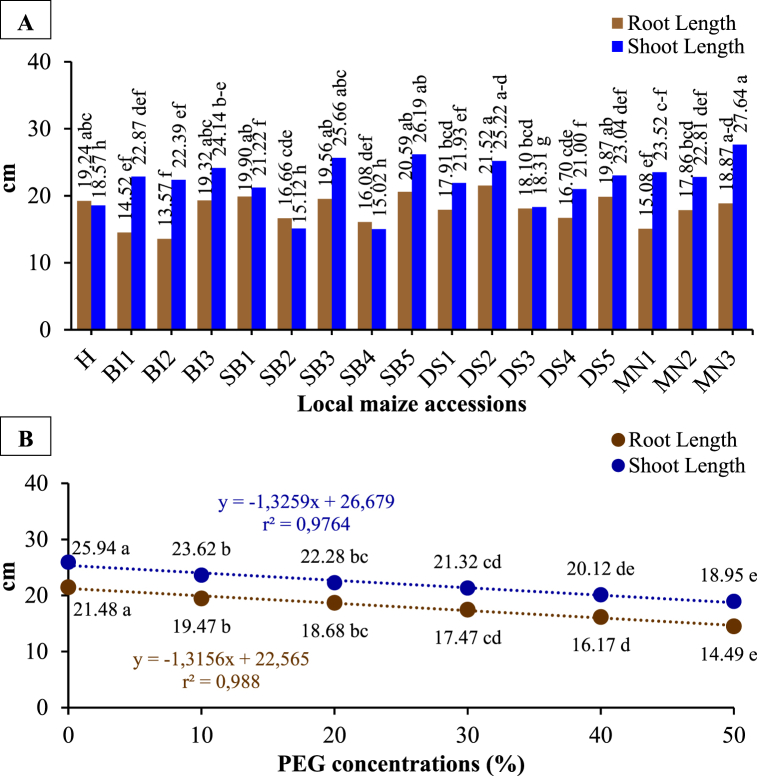
The root and shoot length of local maize from several accessions (A) and PEG concentrations (B). Differences between means for variables with the same letter are not statistically significant according to DMRT test.

There was a significant effect of local maize accessions and PEG concentrations on root and shoot length, however, the interaction effects were not significant (Table 1, Table 2 and Fig. 4(A and B)). A significant decrease in root and shoot lengths was observed by increasing PEG concentrations. Compared to no PEG treatment, DS2 was the most affected accessions in response to the highest PEG concentration (50%), with the largest reduction in root length (13.23 cm) and shoot length (13.20 cm). Conversely, BI2 and MN1 showed the least root and shoot length reductions in response to 50% PEG concentration. The results indicated that PEG concentration had a significant effect on the root and shoot length of the tested accessions. The accessions with the greatest and least response to the applied PEG concentration were DS2 and BI2, respectively.

### 3.4 Root: shoot ratio

**Fig. 5:**
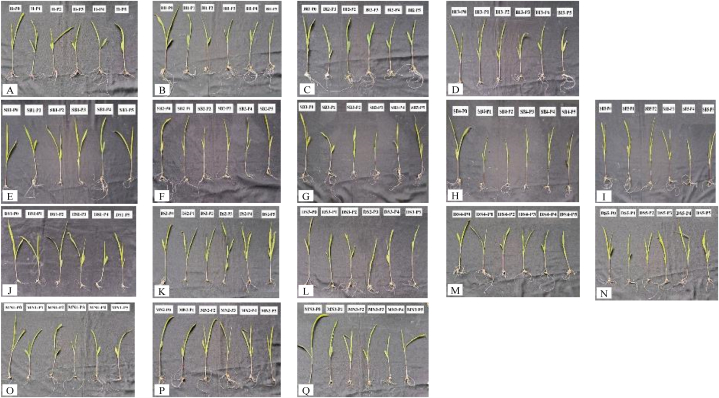
Morphological differences in local maize shoots and roots growth from several accessions and PEG concentrations. Visual analysis of the plants revealed distinct differences in the shoots and roots depending on the accession and PEG concentration. A–D: BI1, BI2, BI3, and SB1 in 5 PEG concentrations. E–I: SB1, SB2, SB3, SB4, and SB5 in 5 PEG concentrations; J–N: DS1, DS2, DS3, DS4, and DS5 in 5 PEG concentrations; O–Q: MN1, MN2, and MN3 in 5 PEG concentrations.

According to ANOVA table (Table 1), there were significant differences between local maize accessions in terms of root: shoot ratio. However, there was no significant effect of PEG concentrations or interactions. SB2 and BI2 accessions had the highest and lowest root: shoot ratios among all accessions, with an average root: shoot ratio of 1.14 and 0.61, respectively (Table 3). Fig. 5 illustrates the morphological differences in the growth of local maize shoots and roots from different accessions and PEG concentrations.

**Table 3:** The root: shoot ratio of local maize from studied accessions, PEG concentrations, and their interaction.

Accessions	PEG concentrations (%)						Average
	0	10	20	30	40	50	
H	1.30	1.15	1.19	0.97	0.90	0.82	1.06 abc
BI1	0.65	0.63	0.66	0.62	0.65	0.61	0.64hi
BI2	0.63	0.63	0.61	0.64	0.62	0.51	0.61i
BI3	0.79	0.85	0.86	0.82	0.81	0.78	0.82 d-h
SB1	0.95	0.90	0.99	0.97	0.97	0.93	0.95b-e
SB2	1.24	1.28	1.04	1.15	1.15	0.96	1.14a
SB3	0.81	0.83	0.82	0.78	0.70	0.65	0.76e-i
SB4	1.00	1.10	1.16	1.16	1.15	1.15	1.12 ab
SB5	0.85	0.81	0.73	0.84	0.78	0.79	0.80e-h
DS1	0.86	0.84	0.84	0.85	0.86	0.74	0.83 d-g
DS2	0.87	0.91	0.87	0.83	0.86	0.76	0.85def
DS3	1.05	0.91	1.07	1.00	1.03	0.98	1.00a-d
DS4	0.70	0.80	0.89	0.90	0.95	0.82	0.85def
DS5	0.94	0.86	0.90	0.92	0.86	0.83	0.88cde
MN1	0.45	0.53	0.72	0.71	0.70	0.77	0.65 ghi
MN2	0.73	0.74	0.82	0.84	0.86	0.81	0.80e-h
MN3	0.81	0.76	0.68	0.62	0.65	0.61	0.69f-i
Average	0.86	0.85	0.87	0.86	0.85	0.80	

Note: Means with the same letters are not significantly different based on Duncan's test at the 5% of probability level.

### 3.5 Fresh weight (g)

**Fig. 6:**
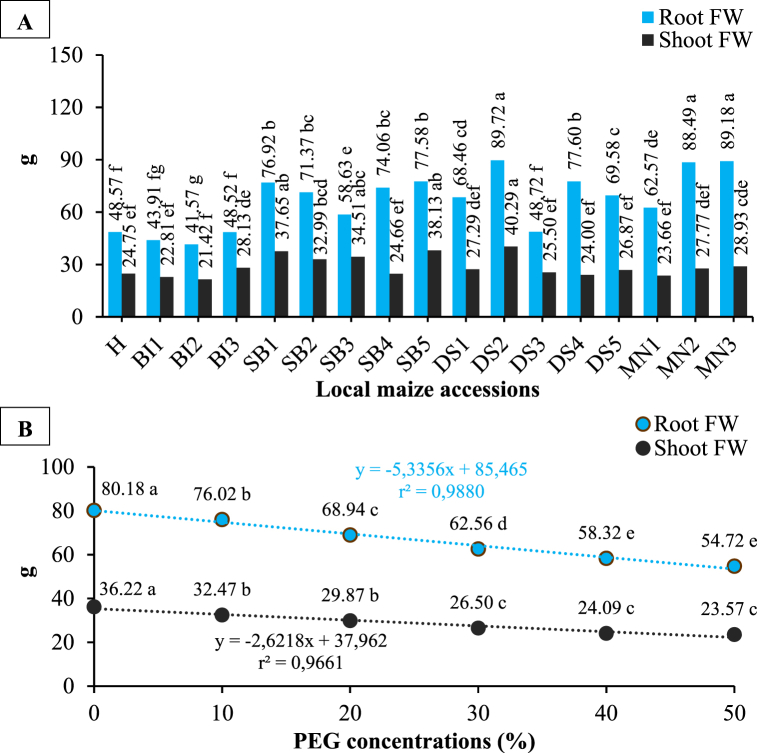
The root and shoot fresh weight of local maize from several accessions (A) and PEG concentrations (B). Differences between means for variables with the same letter are not statistically significant according to DMRT test.

According to ANOVA, fresh root and shoot weights were significantly affected by accessions and PEG concentrations (Table 1 and Fig. 6(A and B)). Meanwhile, the interaction effects (accessions and PEG concentrations) significantly affected the root fresh weight, without significantly affecting the shoot fresh weight (Table 2). BI2 showed the greatest reduction in root fresh weight (52.06 g) in response to high PEG concentration (50%) whereas SB3 showed the largest reduction in shoot fresh weight (24.35 g).

### 3.6 Dry weight (g)

**Fig. 7:**
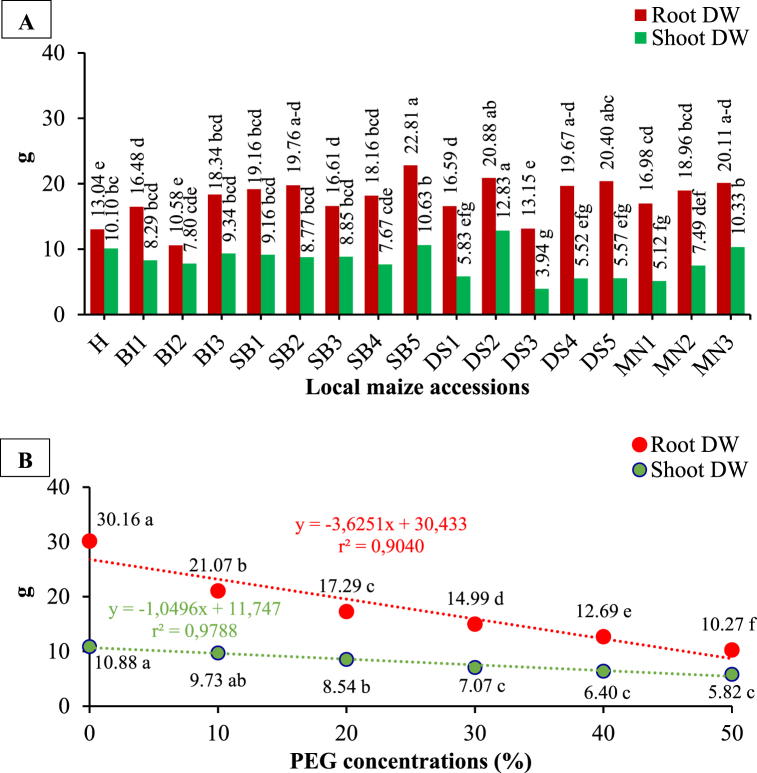
The root and shoot dry weight of local maize from several accessions (A) and PEG concentrations (B). Differences between means for variables with the same letter are not statistically significant according to DMRT test.

As shown in Table 1 and Fig. 7(A and B), accessions and PEG concentrations significantly influenced root and shoot dry weights. Nevertheless, the interaction effects between the accessions and PEG concentrations had a significant impact on root dry weight, but were not significant on shoot dry weight (Table 2). In response to high PEG concentration (50%) SB5 showed the maximum decrease in root dry weight (37.86 g), while DS1 showed the maximum decrease in shoot dry weight (10.6 g).

### 3.7 Water content (%)

**Fig. 8:**
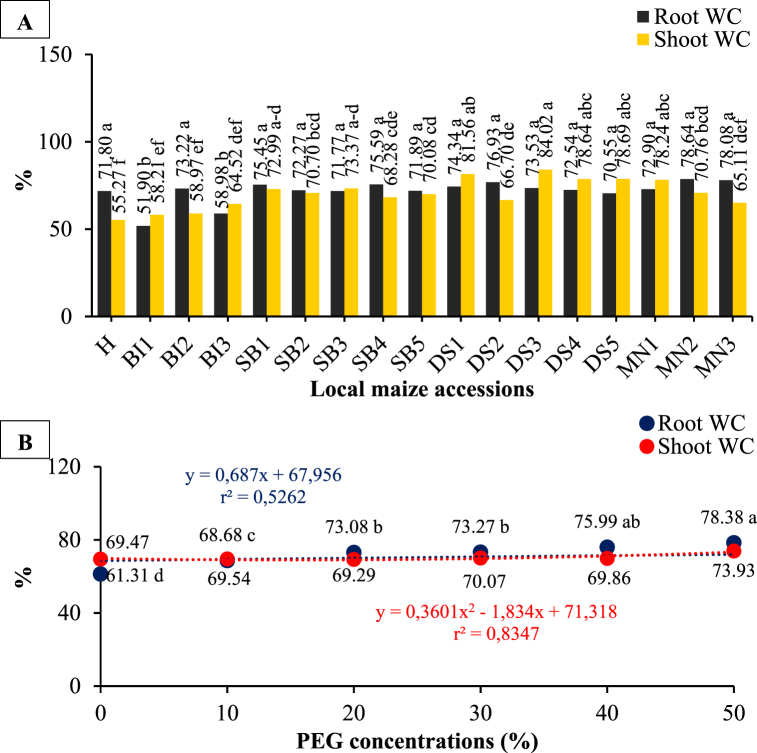
The water content (WC) in the root and shoot of local maize from several accessions (A) and PEG concentrations (B). Differences between means for variables with the same letter are not statistically significant according to DMRT test.

There is a significant difference in root and shoot water content between local maize accessions. Water content in the root increased with PEG concentrations and their interaction with local maize accessions, but had no significant effect on water content in the shoot (Fig. 8(A and B) and Table 1, Table 2). Compared to other accessions, MN2 and DS3 accessions had the highest root and shoot water content (average values) of 78.64 and 84.02% respectively. With high PEG concentration (50%) DS4 showed the greatest increase in root water content (43.83%), while DS1 showed the greatest increase in shoot water content (25.05%). This indicates that the MN2 and DS3 accessions had a higher water content even under normal environmental conditions, which suggests that they have better water retention capabilities. The DS4 and DS1 accessions showed a greater increase in root and shoot water content, respectively, when exposed to high PEG concentration. This suggests that these accessions may be more resilient to drought conditions. However, DTI values might be a more reliable index for detecting drought-tolerant varieties.

### 3.8 Root histological tissue (epidermis, cortex, stele)

**Fig. 9:**
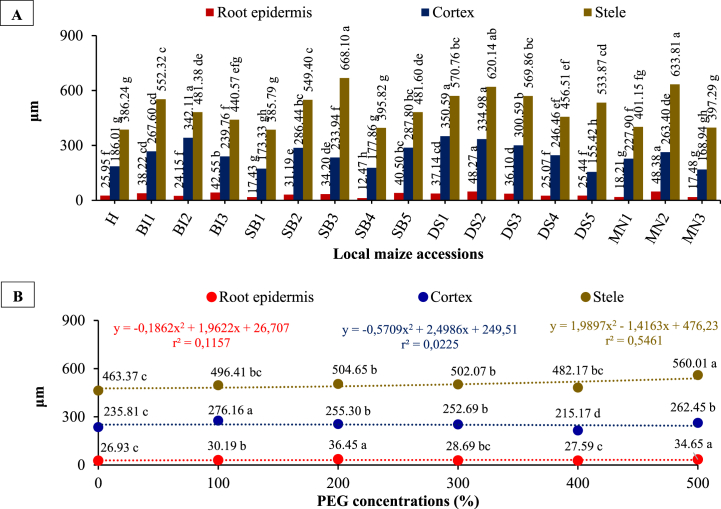
The root epidermis, cortex, and stele size of local maize from several accessions (A) and PEG concentrations (B). Differences between means for variables with the same letter are not statistically significant according to DMRT test.

Local maize root epidermis, cortex, and stele sizes were significantly affected by different accessions, PEG concentrations, and their interactions (Fig. 9(A and B) and Table 1, Table 2). With different concentrations of PEG, MN2 and DS2 accessions showed significantly different sizes of root epidermal tissue (average value) of 48.38 μm and 48.27 μm, respectively. In addition, DS1, BI2, and DS2 accessions had cortex tissue sizes (average values of 350.59 μm; 342.11 μm; and 334.98 μm), whereas SB3 and MN2 accessions had stele tissue sizes with average values of 668.10 μm and 633.81 μm. Generally, five local maize accessions (MN2, DS2, DS1, BI2, and SB3) showed higher histological root size in response to PEG treatments.

**Fig. 10:**
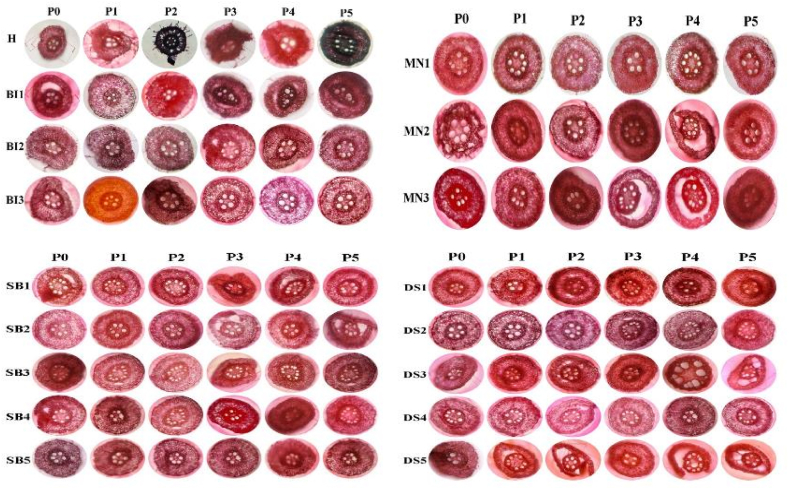
Cross-sections of root epidermis, cortex, and stele of several local maize accessions due to PEG concentrations.

Root and stele epidermal tissue sizes increased at 20% PEG concentrations. Furthermore, tissue size was inhibited up to 40% PEG concentration and then re-boosted at 50% PEG concentration. In similar manner, at 20% PEG, cortex tissue size increased, was inhibited at 40% PEG, and re-boosted at 50% PEG. In general, 50% PEG treatment resulted in an increase in the stele, epidermis, and cortex sizes compared to the control with values of 28.67, 11.30, and 20.85%, respectively. Adaptation to drought stress results in an increase in root tissue size. Fig. 10 shows cross-sections of the root epidermis, cortex, and stele of several local maize accessions exposed to PEG concentrations.

The interaction effect of several accessions with PEG concentrations on germination and root histology of local maize is presented in Table 2. Germination of SB3, SB5, DS2, MN3, and SB1 accessions interaction and without PEG dominantly was greater than that of the other accessions. The DS2 and SB3 interactions with 10% PEG resulted in the highest dry weights of shoots, cortex tissue, and steles. Also, the MN2 interaction with 20% PEG showed the highest root epidermal tissue. In contrast, the hybrid variety with 50% PEG concentration gave the highest root water content.

### 3.9 Heritability value and drought tolerance index (DTI)

The heritability estimation and drought tolerance index of local maize plants from several accessions are shown in Table 4, Table 5. As shown in Table 4 the heritability values (h^2^) of germination and histological characteristics range from 65.05 to 96.41%. There is evidence that the heritability values for all characteristics of local maize depend more on genetics than on environmental factors. Based on Table 5, the hybrid (H) and local accessions (BI3, SB5, DS2, MN3) had the highest DTI values from each location. This revealed that several accessions were tolerant to drought stress during initial selection.

**Table 4:** Estimation of local maize characteristics from several accessions due to PEG concentrations.

Characteristics	σ2g	σ2p	GCV (%)	PCV (%)	h^2^ (%)
Germination					
Germinated seeds	97.02	143.27	121.14	147.21	67.72 (H)
Germination percent	896.79	1159.21	53.39	60.70	77.36 (H)
Vigor index	323.50	374.49	77.68	83.58	86.38 (H)
Root length	25.48	39.17	28.11	34.85	65.05 (H)
Shoot length	73.15	85.12	38.81	41.86	85.94 (H)
Root: shoot ratio	0.14	0.20	43.52	52.47	68.79 (H)
Root fresh weight	1526.79	1619.97	58.50	60.26	94.25 (H)
Shoot fresh weight	179.73	247.05	46.57	54.60	72.75 (H)
Root dry weight	52.30	74.66	40.76	48.69	70.06 (H)
Shoot dry weight	28.90	39.51	66.59	77.86	73.15 (H)
Water content in the root	229.07	346.94	21.08	25.95	66.02 (H)
Water content in the shoot	346.08	559.96	26.44	33.63	61.80 (H)
Histological					
Root epidermis	724.33	758.07	87.52	89.54	95.55 (H)
Cortex	23404.23	24274.47	61.29	62.42	96.41 (H)
Stele	50228.54	57086.04	44.69	47.65	87.99 (H)

Note: heritability criteria (low <20%; moderate = 20–50%; high >50%). Genotypic variance (σ2g); phenotypic variance (σ2p); GCV = genotypic coefficient variance; PCV = phenotypic coefficient variance; h^2^ = heritability.

**Table 5:** The drought tolerance index (DTI) values of local maize plants from several accessions due to PEG concentrations.

Maize accessions	PEG concentrations (%)					Average
	10	20	30	40	50	
H	0.495	0.655	0.690	0.713	0.861	0.683*
BI1	0.315	0.323	0.434	0.617	0.727	0.483
BI2	0.107	0.285	0.441	0.570	0.704	0.421
BI3	0.396	0.401	0.485	0.580	0.660	0.504*
SB1	0.264	0.285	0.414	0.650	0.734	0.469
SB2	0.370	0.492	0.495	0.516	0.602	0.495
SB3	0.280	0.475	0.462	0.327	0.431	0.395
SB4	0.331	0.403	0.473	0.607	0.615	0.486
SB5	0.407	0.529	0.645	0.697	0.806	0.617*
DS1	0.101	0.271	0.113	0.669	0.235	0.278
DS2	0.456	0.476	0.548	0.601	0.743	0.565*
DS3	0.011	0.446	0.564	0.644	0.772	0.487
DS4	0.128	0.414	0.569	0.600	0.624	0.467
DS5	0.333	0.368	0.513	0.603	0.602	0.484
MN1	0.295	0.380	0.488	0.515	0.588	0.453
MN2	0.202	0.426	0.471	0.279	0.518	0.379
MN3	0.290	0.446	0.513	0.555	0.755	0.512*

Note: *indicates drought tolerance of each location.

## 4 Discussion

### 4.1 Effect of local maize accessions

Germination is one of the most crucial stages in its life cycle, so drought stress is especially important during this phase. As a result of their high heritability, MN3, SB2, DS3, DS1, and SB3 accessions have the largest shoot length, root: shoot ratio, and water content in the shoot, cortex, and stele (Table 4). These traits are important for plants to survive in drought conditions. Therefore, these accessions are more suitable for drought-prone areas and can be used for further breeding programs. This high heritability indicates that these characteristics are predominantly influenced by genetic factors and can be passed down from generation to generation. As Fehr [24] points out, a high heritability value implies that genetic factors play a more significant role in the appearance and character measure that can be inherited. This knowledge can be used to better understand the origin of these characteristics and to identify potential strategies for their modification. Additionally, it can provide insight into the effects of environmental influences on the traits.

It is considered that the stress index of dry weight is a reliable indicator of a plant's exposure to stress of any kind [25]. Osmotic stress causes low water availability, resulting in decreased cell division and elongation. This is due to a decrease in turgor pressure and cell growth. Thereafter, biomass and particularly dry weight are reduced [26]. According to Badr et al. [27], 40 maize accessions treated with 10% PEG yielded high heritability in seedling characteristics such as germination percent, root and shoot length, fresh weight, and dry weight. Under drought stress conditions, Khan et al. [28] also determined seedling characteristics such as germination percent, root and shoot length, fresh and dry weight of 40 different maize varieties with relatively high heritability, ranging from 62.09 to 89.56%. Furthermore, the study also suggested that the heritability of the seedling characteristics was largely dependent on the type of maize variety and the severity of drought stress. However, it is imperative to note that our study was conducted under controlled conditions. The study results may not be representative of what would happen in a natural environment where conditions are not as controlled. As such, further studies should be conducted in a natural environment and under field conditions to confirm the findings of the study. In addition, further studies should explore the impacts of other environmental factors on seedling characteristics heritability.

### 4.2 Effect of PEG concentrations

Seed germination in this study was affected by drought stress. However, stress intensity depended on genetics and PEG concentrations (Table 1, Table 2). As an osmotic agent, PEG 6000 is used in these experiments. In addition to mineral elements, hormones, and protein metabolism, PEG also regulates signal transmission [25]. Seed germination is inhibited by PEG because it slows down seeds' moisture rate [29]. In general, higher PEG concentrations inhibited germination and histological characteristics of local maize from North Sumatra. It was due to PEG's interference with water absorption and inhibits plant germination. This is because PEG molecules are hydrophilic, meaning they attract water molecules and absorb them, thus preventing the seed from receiving the necessary moisture for germination. Additionally, PEG molecules interfere with signal transmission, which can prevent the seed from receiving the necessary chemical signals for germination. As such, PEG can have a detrimental effect on seed germination, as it not only absorbs water from the environment, but also prevents other necessary signals for germination. In several studies, similar findings have been reported regarding germination and seedling parameters. It has been found that drought stress reduces the water potential gradient in seeds in several studies, including Ajirloo et al. [30]; Boureima et al. [31]; and Channaoui et al. [32]. As a result, water movement and absorption are reduced and seed germination is delayed. Additionally, Khan et al. [33] found that drought stress inhibits germination through the release of reactive oxygen species (ROS) that damage cell structures and metabolic processes. Drought stress was associated with a delay in germination due to decreased water uptake, low energy supply, and disruptions in enzymatic activity, according to Okçu et al. [34]. All of these effects of drought stress can lead to significant decreases in crop yields and increases in food insecurity, particularly in areas where droughts are frequent. In order to reduce the impact of drought stress, it is important to use efficient irrigation methods and drought-tolerant crop varieties.

Multiple studies have reported delayed germination due to PEG disruption of physiological and biochemical processes. With an increase in PEG concentrations up to 20%, Hellal et al. [35] reported a decrease in water content in roots and shoots, vigor index, germination percent, and fresh and dry weights of barley plants, which is similar to the findings of our study. These findings demonstrate the negative impacts of PEG on germination and plant growth, highlighting the need for further research on sustainable agricultural practices. result of drought stress in *Sorghum* caused by PEG, Zhang et al. [36] found inhibition of germination percent, vigor index, relative water and chlorophyll content, and decreased malondialdehyde. However, plant antioxidants like ascorbate peroxidase, catalase, peroxidase, and superoxide dismutase increased. Reduced cell division and plant growth metabolism could be responsible for these reduced germination characteristics [37]. It has been reported by Batool et al. [38] that 15% PEG treatments to sensitive and tolerant plants resulted in a decrease in chlorophyll (a, b, total), carotenoids, and relative water content, but an increase in enzymes such as ascorbate peroxidase, catalase, peroxidase, and superoxide dismutase. Magar et al. [11] also found that PEG treatment up to −15 bars decreased germination percent, root and shoot length, fresh and dry weight, and vigor index of seedling maize. With an increase in PEG to 6%, Asghar et al. [39] observed a decrease in relative water content, chlorophyll *a* and *b*, photosynthetic rates, and stomatal conductance. These findings demonstrate that even mild levels of osmotic stress can influence physiological processes and reduce the overall growth and development of seedlings. This is important for understanding the effects of drought on crop production, as well as how changes in climate can affect agricultural systems.

PEG concentration significantly affects the histological size changes of root tissues such as the epidermis, cortex, and stele. There was an increase in the roots histological size by 28.67; 11.30; and 20.85% due to 50% PEG treatment compared to the control (Fig. 9(B)). The increase in root tissue size is local maize as an adaptation form to protect the roots against drought stress. The phenomenon of root anatomy tissue is closely related to increasing water retention by the roots. The increased root tissue size was also correlated with higher degree of water absorption by the roots, which can help in improving the plant's drought tolerance. The results of this study suggest that PEG can be used to improve root tissue size and water uptake in maize to increase drought tolerance. This finding could be applied to other crops as a way of improving drought resistance, and could help to improve yields in dry climates. These findings are supported by Lynch [40] and Melo et al. [41] who reported that root anatomy will affect the efficiency of water absorption and storage in the roots and the cortex thickness can increase plant tolerance to drought stress. Rosawanti et al. [42] found an increase in the cortex thickness and stele diameter of soybean genotype SC39-1 caused by 20% PEG treatment compared to control. Similarly, Melo et al. [41] revealed that the root epidermis thickness and cortex of Arabica coffee increased during drought conditions compared to control. Pereira et al. [43] added that cortex thickness is closely related to hydraulic conductivity. This is because the lower the cortex thickness, the higher the hydraulic conductivity, which can modify the roots to absorb water in drought conditions. Additionally, Makbul et al. [44] reported an increase in soybean plants' cortex diameter as an adaptation to drought stress. This higher cortex thickness helps protect the root from the harsh environmental conditions, such as drought, as it allows for a greater absorption of water and other nutrients. This is advantageous for the plant as it helps to ensure its survival. As such, this adaptation serves as an effective method to increase the plant's capability of surviving drought conditions.

### 4.3 Interactions effect of accessions with PEG concentrations

Our findings revealed that the interaction between different accessions and PEG concentrations of 0–10% gave more favorable germination and histological characteristics compared to those with higher PEG concentrations. According to Table 3, genetic factors dominated germination and histological characteristics of local maize with a heritability of 65.05–96.41%. However, PEG treated up to 50% inhibited germination characteristics and increased root tissue histological size. Dry and fresh biomass and root and shoot length were negatively impacted by drought stress induced by PEG. There is a possibility that water stress could affect the development of root cells, decreasing nutrient uptake as a result. Therefore, further research is necessary to understand the exact mechanisms of water stress on maize growth and development. This could help to better predict the effects of water stress on crop production and to develop strategies for improving crop resilience in water-limited environments. In turn, this adversely affects photosynthesis, which is crucial to biomass production and the development of roots and shoots [45].

Based on the heritability value and drought tolerance index (Table 4, Table 5), local maize accessions, namely BI3, SB5, DS2, and MN3 from North Sumatra were classified as tolerant to drought stress by PEG-6000 induction. This result also showed that the increase in root anatomical tissue size such as the epidermis, cortex, and stele due to PEG-6000 can be used as a reference for root protection against drought stress, which is called an adaptation mechanism. These maize accessions also had a higher heritability value which meant that their drought tolerance is likely to be passed down to their offspring. The increase in root anatomical tissue size due to PEG-6000 can also help reduce water loss from the roots and help the plants retain more water, thus increasing their drought tolerance. This result is still an initial selection in the early stages of plant growth, hence, it is necessary to conduct further tests to determine the drought tolerance level through field capacity testing. These findings also support the idea that the increase in root tissue size is an adaptation mechanism that can protect against drought stress, and that further research is needed to determine how effective this adaptation is in long-term and actual conditions.

## 5 Conclusions

From the aforementioned research findings, it can be concluded that PEG-induced drought stress has a distinct effect on maize seed germination and seedling growth. The local maize accessions BI3, SB5, DS2, MN3, and a hybrid variety (H) responded favorably under drought conditions and hence can be declared drought tolerant. In contrast, DS1, MN2, SB3, and BI2 local maize accessions were regarded as drought sensitive. In local maize, PEG concentrations up to 50% significantly inhibited germination parameters. However, a high concentration of PEG increases the histological characteristics of the roots, such as the epidermis, cortex, and stele. The study also revealed that the drought tolerance index (DTI) was a reliable indicator for identification of drought tolerant maize. Therefore, BI3, SB5, DS2, MN3 could be considered as parents for drought-tolerant varieties or direct cultivation. Drought-tolerant maize varieties have potential implications for crop productivity, food security, and sustainable agriculture, especially in the face of climate change. The results of this study can be used to develop efficient strategies for drought-tolerant maize breeding. Also, the findings can be applied to improve maize drought tolerance in arid and semi-arid regions. This would help farmers to increase their yields and reduce losses from droughts. It would also lead to more efficient use of resources, such as water, enabling farmers to optimize production while reducing environmental impact. Additionally, it could lead to improved food security in drought-prone regions. For the findings of this study to be confirmed in the field, further research is needed. Additionally, the effects of PEG-induced drought stress on other maize varieties should also be explored. Finally, future studies should consider drought stress effects on a larger scale. Such studies should take into account the effects of environmental factors such as temperature, soil composition, and light intensity on the various aspects of drought stress.

## Funding

This research is funded by Ministry of Education, Culture, Research, and Technology, (Indonesia) with the number 153/E5/PG.02.00.PT/2022.

## Author contribution statement

Conceived and designed the experiments: Novilda Elizabeth Mustamu; Koko Tampubolon; Alridiwirsah Alridiwirsah.

Performed the experiments: Novilda Elizabeth Mustamu; Koko Tampubolon; Mohammad Basyuni.

Analyzed and interpreted the data: Novilda Elizabeth Mustamu; Alridiwirsah Alridiwirsah; Mohammad Basyuni; Mehdizadeh, M.; Duraid K.A. AL-Taey; AL Janabi, Haider Jawad Kadhim AL-Janabi.

Contributed reagents, materials, analysis tools or data: Novilda Elizabeth Mustamu; Koko Tampubolon; Alridiwirsah Alridiwirsah.

Wrote the paper: Novilda Elizabeth Mustamu; Koko Tampubolon; Alridiwirsah Alridiwirsah; Mohammad Basyuni; Mehdizadeh, M.; Duraid K.A. AL-Taey; AL Janabi, Haider Jawad Kadhim AL-Janabi.

## Data availability statement

Data included in article/supp. material/referenced in article.

## Additional information

No additional information is available for this paper.

## Declaration of competing interest

The authors declare that they have no known competing financial interests or personal relationships that could have appeared to influence the work reported in this paper.
